# The Impact of Reducing Psychiatric Beds on Suicide Rates

**DOI:** 10.3389/fpsyt.2019.00448

**Published:** 2019-07-02

**Authors:** Jo-An Atkinson, Andrew Page, Adam Skinner, Mark Heffernan, Ante Prodan, Ian B. Hickie

**Affiliations:** ^1^Decision Analytics, Sax Institute, Sydney, NSW, Australia; ^2^Translational Health Research Institute, Western Sydney University, Sydney, NSW, Australia; ^3^Dynamic Operations, Sydney, NSW, Australia; ^4^School of Computing, Engineering & Mathematics, Western Sydney University, Sydney, NSW, Australia; ^5^Brain and Mind Centre, University of Sydney, Sydney, NSW, Australia

**Keywords:** public mental health, suicide prevention, threshold effects, psychiatric beds, systems modeling and simulation

## Abstract

There has been ongoing debate regarding the impact of reductions in psychiatric beds on suicide rates, and the potential effect of reallocation of acute hospital funding to community-based mental health programs and services. Computer simulation offers significant value in advancing such debate by providing a robust platform for exploring strategic resource allocation scenarios before they are implemented in the real world. We report an application that demonstrates a threshold effect of cuts to psychiatric beds on suicide rates and the role of context specific variations in population, behavioral, and service use dynamics in determining where that threshold lies. Findings have important implications for regional decision-making regarding resource allocation for suicide prevention.

## Introduction

The past decade has seen debate regarding the impact of reductions in psychiatric beds on suicide rates and the potential effect of reallocation of acute hospital funding to community-based psychosocial, primary, and community health services (1–5). The debate centers around the key questions of whether there is a threshold at which reductions in beds start to adversely impact suicide rates, where that threshold lies, and therefore the minimum number of beds per 100,000 population required. The answers remain elusive and difficult to study in the real world.

Computer simulation offers value allowing exploration of likely impacts of counterfactual scenarios in a low-risk way. An existing systems model developed for decision analysis to support regional suicide prevention planning (6) was used to answer three questions: i) Do all cuts to psychiatric beds increase suicides? ii) If there is a threshold, where does it lie? iii) Can the threshold be raised by increasing capacity of community-based services?

## Methods

A system dynamics model was developed for Western Sydney Primary Health Network (PHN) (6). Western Sydney is one of the fastest-growing urban populations in Australia with a diverse population and geography. The structure, parameterization, calibration, and validation of the model drew on a range of data sources including population survey data, systematic reviews (and meta-analyses), administrative data, and expert knowledge of multidisciplinary stakeholders, detailed elsewhere (6). Briefly, the model captures i) the pathway from vulnerability, to psychological distress, to mental disorder; ii) mental health service pathways, including assessment of care needs and delivery of low-to-moderate-intensity services (community-based services), and high-intensity services (tertiary or hospital services); iii) pathways to suicidal behavior (attempted suicide and suicide) either with or without contact with mental health services; and iv) mental health recovery pathways with or without contact with mental health services. The model has an open population, with births and migration contributing to the population and deaths (from causes other than suicide) subtracting from the population. The computational model was developed using Stella Architect^®^ software (www.iseesystems.com/). Estimates of the effects of interventions, and the specific mechanism of action in the model, were based on evidence from the literature and informed by stakeholder feedback for local application. Key assumptions of the model are provided in **Supplementary file 1**, and all model parameters are published in Page et al. (6).

The effect of tertiary service capacity on suicide was explored by simulating a range of scenarios in which the number of psychiatric beds per 10^5^ population was reduced at the start of 2018, either with or without a simultaneous increase in community-based services capacity (the number of community-based practitioners per 10^5^ population). For each scenario, we compared the cumulative number of suicides for the period 2018–2028 with the corresponding number of suicides under a baseline scenario of no change in psychiatric beds or community-based services capacity. The impact of uncertainty in the estimates on the simulation results for two key model parameters that determined the rate at which patients enter mental health service pathways (general practice visits per person per year and psychiatric assessment capacity per week) was assessed *via* sensitivity analysis. Latin hypercube sampling was used to draw 100 sets of values for each parameter from a uniform joint distribution spanning ±10% of the default values. Differences in projected suicides between the baseline and services capacity reduction scenarios were calculated for each set of parameter values and summarized using simple descriptive statistics.

## Results

**Figure 1:**
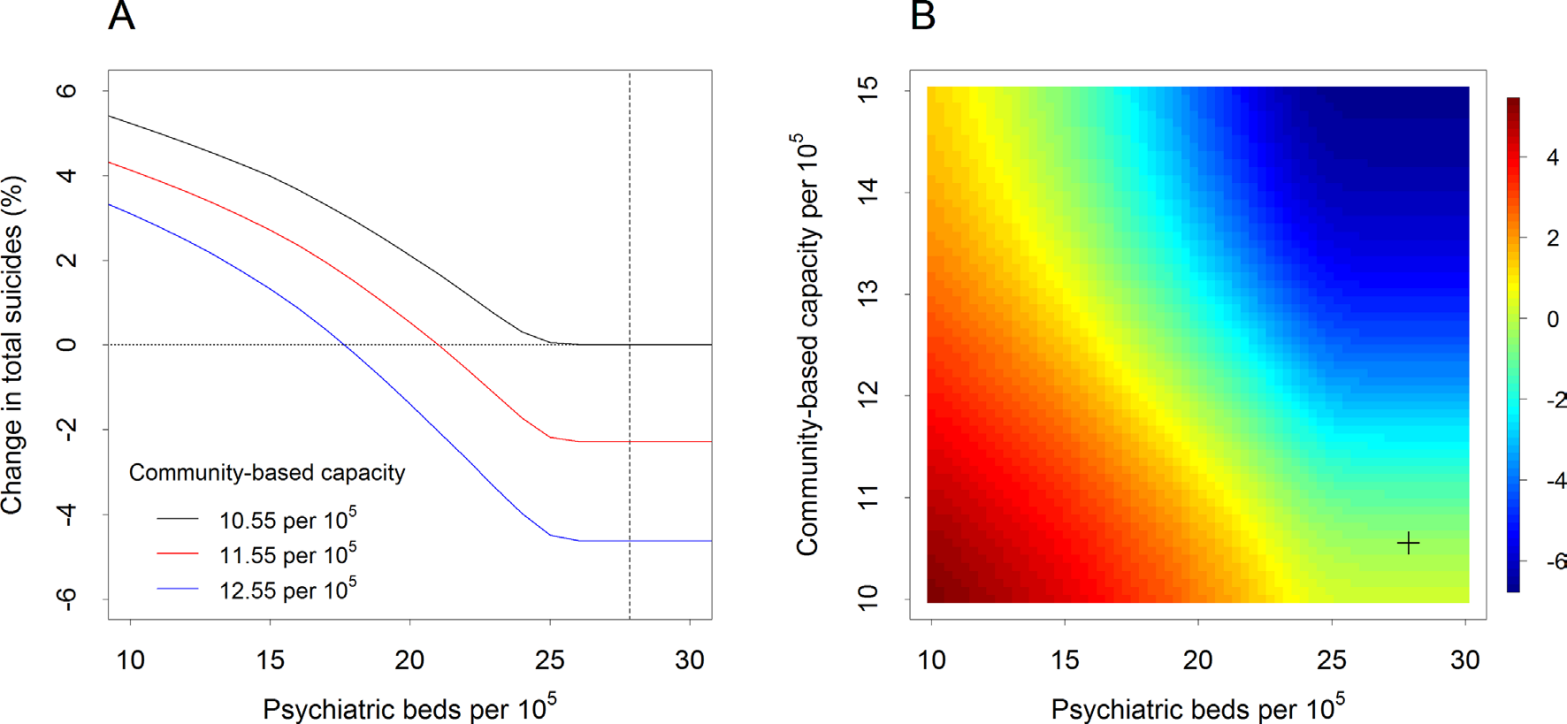
Impact of changes in psychiatric bed availability and community-based mental health service capacity on forecast percentage changes in suicide (Western Sydney, 2018–2028) *(A) Change in the total number of suicides over the period 2018–2028 compared with the baseline scenario (expressed as a percentage of the baseline total) as a function of the number of psychiatric beds per 10^5^ population assuming different levels of non-secondary services capacity (i.e., numbers of community-based practitioners per 10^5^ population). The dashed vertical line indicates the baseline number of psychiatric beds per 10^5^ population. **(B)** Percentage change in the number of suicides (compared with the baseline total) as a function of secondary and non-secondary services capacity. The cross in the lower right corner indicates the baseline capacity values (27.86 psychiatric beds and 10.55 community-based practitioners per 10^5^ population).

Under the baseline scenario, in which capacity was maintained at 27.86 psychiatric beds and 10.55 community-based practitioners per 10^5^ population, 1,862 suicides (**Table 1**) in the Western Sydney catchment were forecast for the period 2018–2028. With no increases in the community-based service workforce, reductions in psychiatric beds to less than 26 beds per 10^5^ population increased suicide deaths compared with the baseline (**Figure 1**). Under scenarios in which community-based service capacity was increased by one to two practitioners per 10^5^ population, the threshold number of psychiatric beds that could be cut without adversely impacting suicide deaths increased from approximately 2 per 10^5^ (with no additional community capacity) to 6–10 per 10^5^ (i.e., the number of psychiatric beds could be reduced to 18–22 per 10^5^ without increasing the cumulative number of suicides relative to the baseline scenario).

**Table 1:** Sensitivity analysis results.

Psychiatric beds per 10^5^	Suicides (median)	Change in total suicides (%)		
		Median	2.5th pctl	97.5th pctl
Baseline (10.55 community practitioners per 10^5^)				
27.86	1,862	–	–	–
Community capacity per 10^5^ = 10.55				
10	1,963	5.40	2.98	8.30
15	1,938	4.15	1.94	6.68
20	1,903	2.22	0.60	4.04
25	1,866	0.05	0	0.69
30	1,862	0	−0.02	0
Community capacity per 10^5^ = 11.55				
10	1,942	4.23	2.30	6.87
15	1,913	2.84	1.16	4.77
20	1,876	0.44	−0.31	1.53
25	1,826	−1.90	−3.98	−1.01
30	1,820	−2.29	−4.11	−1.01
Community capacity per 10^5^ = 12.55				
10	1,921	3.19	1.74	5.17
15	1,889	1.40	0.47	2.23
20	1,840	−1.34	−2.91	−0.42
25	1,786	−4.12	−8.11	−1.73
30	1,779	−4.42	−8.26	−1.73

*Numbers of suicides for the period 2018–2028 and change in the number of suicides compared with the baseline (expressed as a percentage) for varying numbers of psychiatric beds and community-based practitioners per 10^5^ population. Medians and percentiles (pctl) are based on 100 simulations (see text).

## Discussion

These findings suggest that i) not all reductions to psychiatric beds will result in increases in suicide deaths; ii) a threshold appears to exist beyond which cuts are likely to adversely impact suicides; and iii) the threshold can be significantly increased by strengthening community-based mental health services capacity. Given the moderating effect of community-based mental health services, setting a “safe minimum number of psychiatric beds” (3) cannot be standardized. Context-specific variations in population, behavioral, and service use dynamics will determine where a threshold lies and the extent to which reallocation of acute hospital funding to community-based psychosocial, primary, and community health services will deliver improved suicide prevention outcomes. Such dynamics are best captured using systems modeling approaches that deliver useful decision analytic tools to support service planning for suicide prevention. Given the implications of service balance this study has highlighted, regional level systems modeling and simulation should be undertaken to test service planning scenarios before they are implemented in the real world.

## Author Contributions

Manuscript concept, design, and drafting: JA. Data acquisition, analysis, model development: AP, JA, MH, IH, AP. Statistical analysis: AS, JA. Critical revision of manuscript for important intellectual content: all authors.

## Funding

This article is based on research funded by a Western Sydney University Partnership Grant with in-kind contributions from WentWest—Western Sydney Primary Health Network (in part with the Commonwealth Department of Health), and Western Sydney Local Health District.

## Conflict of Interest Statement

The authors declare that the research was conducted in the absence of any commercial or financial relationships that could be construed as a potential conflict of interest.

## Supplementary Material

Supplementary File 1 Key model assumptions.
