# Supplementary material for: The Impact of Reducing Psychiatric Beds on Suicide Rates
Source: Front Psychiatry. 2019 Jul 2;10:448. doi: 10.3389/fpsyt.2019.00448 (PMC6615492; doi:10.3389/fpsyt.2019.00448)
Supplement: Supplementary File 1 — Key model assumptions. [file DataSheet_1.pdf]

Supplementary file 1: Key model assumptions

Key assumptions regarding the core component of the dynamic model are highlighted below:

- The rate at which the general population of Western Sydney become vulnerable is 2% per annum.
- The rate at which the vulnerable subset of the population become distressed is twice that of the general population in Western Sydney;
- The risk of suicide attempts among those in the 'Distressed or active disorder' state is significantly greater than the risk among the general population (0.4% vs 0.025%);
- For simplicity in the model there are no suicides among those in active treatment;
- All-cause mortality rate among the 'vulnerable' and 'distressed or active disorder' subpopulations are slightly higher than the general population.

Key assumptions / default parameters for the service capacity interventions tested:

- **Community-based or primary care service capacity** refers to the number of practitioners available to deliver low to moderate intensity mental health services. These practitioners include community-based psychiatrists, psychologists, social workers and mental health nurses. In the model, as service capacity is increased, the average waiting time for services decreases; with the intervention acting to increase the flow between the stock of people waiting for community-based mental health services and those receiving services. Model estimates for this intervention are based on data provided by WentWest - Western Sydney Primary Health Network and Western Sydney Local Health District.

Default assumptions: The simulations assume delivery of an average of 5 service contacts per patient with service interval of 4-weeks, and a baseline capacity of 10.55 practitioners per 10^5 population.

- **Tertiary mental health service capacity** refers to the number of public psychiatric hospital beds available. In the model, increasing the number of hospital beds available acts on the flow between the stock of people waiting for tertiary mental health services and those admitted to services. Model estimates for this intervention are based on data provided by WentWest - Western Sydney Primary Health Network and Western Sydney Local Health District, on number of current psychiatric beds available and average length of stay.

Default assumptions: The simulations assume a baseline capacity of 27.86 beds per 10^5 population and an average length of stay of 3.5 weeks.
